# A primary gastric synovial sarcoma: A case report and literature review: Erratum

**DOI:** 10.1097/MD.0000000000009742

**Published:** 2018-01-26

In the article, “A primary gastric synovial sarcoma: A case report and literature review”^[1]^, which appeared in Volume 96, Issue 49 of *Medicine*, the authors would like to make several corrections.

The order of the authors should be “In Tae So^a^, MD, Kwang-Bum Cho^a∗^, MD, PhD, Yu Na Kang^b∗^, MD, PhD, Ju Yup Lee^a^, MD, Sang Jin Kim^a^, MD, Hye In Jung^a^, MD, Jong-Hwan Choi^a^, MD, Yoo Jin Lee^a^, MD, Hyun Jik Lee^a^, MD, Kyung Sik Park^a^, MD, PhD, Seung Wan Ryu^c^, MD, PhD.”

The affiliations should be “^a^Department of Internal Medicine, Keimyung University School of Medicine, Daegu, Korea, ^b^Department of Pathology, School of Medicine, & Institute for Cancer Research, Keimyung University, Daegu, Korea, ^c^Department of Surgery, Keimyung University School of Medicine, Daegu, Korea.*”*

The corresponding author's information should be “^∗^Correspondence: Kwang Bum Cho, Yu Na Kang, 56 Dalseong-Ro, Jung-gu, Daegu 41931, South Korea (e-mail: chokb@dsmc.or.kr; yunakang@dsmc.or.kr).”

The abstract should appear as:

**Rationale:** When a gastric spindle cell tumor is observed, the possibility of synovial carcinoma, besides common mesenchymal tumor, should also be considered.

**Patient concerns:** The patient is a 51-year-old American woman who underwent medical check-up at a general hospital. Upper endoscopy showed a 2-cm sized mass covered with intact mucosa, and a central depression located on the posterior wall of the mid body.

**Diagnoses:** Biopsy of the mass showed focal atypical cells proliferation in mucosa on hematoxylin & eosin (H&E) staining. Endoscopic ultrasound showed a 17-mm homogenously hypoechoic mass within the submucosal layer. After diagnostic endoscopic submucosal dissection was performed, H&E and immunohistochemical staining showed synovial sarcoma (SS). To confirm the diagnosis, reverse transcriptase-polymerase chain reaction was performed, revealing a chimeric transcript of the SYT-SSX1 fusion gene. The diagnosis of primary gastric SS was confirmed because no evidence of possible primary lesions or metastatic lesions was observed.

**Interventions:** The patient underwent distal gastrectomy.

**Outcomes:** After surgery, the surgical specimen demonstrated no residual tumor cells. The patient received no adjuvant therapy, and there has been no evidence of local recurrence or distant metastasis for 2 months after the operation.

**Lessons:** When gastric subepithelial tumor is suspicious, we should also consider gastric SS.

**Abbreviations**: ESD = endoscopic submucosal dissection, RT-PCR = reverse transcriptase-polymerase chain reaction, SS = synovial sarcoma.

**Keywords:** endoscopy, gastric neoplasm, synovial sarcoma

The following funding information should appear:

Funding: This work was supported by the research promoting grant from the Keimyung University Dongsan Medical Center in 2005 and 2013. The funders had no roles in study design, data collection and analysis, preparation of the manuscript, or decision to publish. The corresponding authors had full access to all the data in the study and had final responsibility for the decision to submit for publication.
